# Seed-Specific Stable Expression of the α-AI1 Inhibitor in Coffee Grains and the *In Vivo* Implications for the Development of the Coffee Berry Borer

**DOI:** 10.1007/s12042-015-9153-0

**Published:** 2015-10-08

**Authors:** Érika V. S. Albuquerque, Caroline A. Bezerra, Juan V. Romero, Jorge W. A. Valencia, Arnubio Valencia-Jiménez, Lucas M. Pimenta, Aulus E. A. D. Barbosa, Maria C. M. Silva, Ana M. Meneguim, Maria Eugênia L. Sá, Gilbert Engler, Janice de Almeida-Engler, Diana Fernandez, Maria F. Grossi-de-Sá

**Affiliations:** Embrapa Recursos Genéticos e Biotecnologia, 70770-917 Brasília, DF Brazil; Universidade Católica de Brasília, 70790-160 Brasília, DF Brazil; Facultad de Ciencias Agropecuarias, Universidad de Caldas, Apartado aéreo 275, Manizales, Colombia; Universidad del Atlántico, km 7, Barranquilla, Colombia; IAPAR, Instituto Agronômico do Paraná, 86047-902 Londrina, Brazil; EPAMIG, Empresa de Pesquisa Agropecuária de Minas Gerais, 38001-970 Uberaba, MG Brazil; INRA, Institut National de la Recherche Agronomique, Plant, Health and Environment, Plant-Nematodes Interaction Team, UMR 1355 ISA/Centre National de la Recherche Scientifique, Sophia-Antipolis, France; IRD, Institut de Recherche pour le Développement UMR 186 - IRD/CIRAD/UM2 Résistance des Plantes aux Bio-agresseurs, 34394 Montpellier Cedex 5, France

**Keywords:** Fruit-specific expression, *Hypothenemus hampei*, Genetically modified plants, *Coffea arabica*, Immunolocalization

## Abstract

Genetic transformation of coffee (*Coffea* spp.), the second most traded commodity worldwide, is an alternative approach to introducing features that cannot be introgressed by traditional crossings. The transgenic stability, heritability and quantitative and spatial expression patterns of the seed-specific promoter phytohemagglutinin (PHA-L) from *Phaseolus vulgaris* were characterized in genetically modified *C. arabica* expressing the α-amylase inhibitor-1 (*α-AI1*) gene. The α-AI1 inhibitor shows considerable activity toward digestive enzymes of the coffee berry borer (CBB) *Hypothenemus hampei*. This insect pest expends its life cycle almost entirely in coffee berries. Transgene containment in the fruit is important to meeting food and environmental safety requirements for releasing genetically modified (GM) crops. PCR analysis of T2 coffee plants showed a Mendelian single-copy segregation pattern. Ectopic transgene expression was only detected in coffee grains, as demonstrated by reverse transcription-PCR analysis of different plant tissues. An intense immunocytochemical signal associated with α-AI1 protein expression was localized to endospermic cells. In addition, a delay in the larval development of CBB was observed after challenging transgenic coffee seeds with the insect. These results indicate that the PHA-L promoter might be a useful tool in coffee for the seed-specific expression of genes related to coffee bean productivity, quality and pest protection. The biotechnological applicability of the *α-AI1* gene for controlling CBB is also discussed. This work is the first report showing a seed-specific transgene expression in coffee plants.

**Electronic supplementary material:**

The online version of this article (doi:10.1007/s12042-015-9153-0) contains supplementary material, which is available to authorized users.

## Introduction

Coffee is a favorite beverage worldwide, and the coffee international market provides economic support to many coffee-producing countries. *Coffea arabica* and *Coffea canephora*, the two most commercialized species (ICO 2014), are extremely vulnerable to damage caused by *Hypothenemus hampei* (Ferrari) (Coleoptera: Scolytidae), also known as the coffee berry borer (CBB). Although endemic to Africa, the CBB is broadly distributed worldwide and responsible for considerable economic impacts not only on yield and quality but also on the environment (Baker et al. (2002). This problem is forecasted to worsen in climate change scenarios where the calculated hypothetical number of generations per year of *H. hampei* is predicted to increase in all *C. arabica*-producing areas from five to ten (Jaramillo et al. 2011). Recent CBB invasions have even threatened coffee crops on Hawaiian farms by Burbano et al. (2011). A study of the economic damage caused by the CBB in Brazilian coffee fields using attraction traps (Fernandes et al. 2011) revealed that the quantitative losses caused by *H. hampei* ranged from 7.9 to 23.7 % of bored berries for high- and average-yield conventional crops, respectively, whereas in organic coffee, 24.4 to 47.6 % of berries, respectively, were bored.

Of all *Hypothenemus* species, *H. hampei* is the most studied due to the worldwide damage it causes to coffee grains, affecting both yield and grain quality. Nevertheless, a recent review of the literature published on the CBB indicates that research outputs are not what would be expected for such an economically relevant commodity as coffee (Vega et al. 2015a). In general, the strategies to control CBB adults have mainly focused on the use of pesticides, biological products with insecticidal activity and crop management activities, as adopted in integrated management programs (Damon 2000; Jaramillo et al. 2006). Numerous strategies have been described for CBB control, including the use of Bethylidae wasps that parasitize *H. hampei* (reviewed by Bustillo 2002); the selection of *H. hampei-*resistant *Coffea* germplasm via an antibiosis test (Álvarez et al. 2001); studies of secondary metabolites from entomopathogenic fungi (Valencia 2011); integrated pest management programs (Bustillo et al. 1998); and Bt genes from *Bacillus thuringiensis* serovar *israelensis*, which is highly toxic to the first instar larvae of the CBB (Méndez-López et al. 2003). However, the CBB’s life cycle occurs almost entirely in coffee seeds, making the use of chemicals not only difficult but also inefficient (Bustillo 2002). The small CBB female lays multiple eggs inside the coffee berry, which hatch into larvae that feed upon the coffee seeds (seeds that later will develop into the beans) inside the berry. After the pupae stage, the CBB adults emerge inside the berry, where mating occurs. Only the females have functional wings that allow them to search for new coffee berries to infest during a very short period outside the fruits (Damon 2000).

Like other insects, the CBB adult contains several *α-*amylases that are used to break down starch-containing seeds for its development (Baker 1983). In 2000, Valencia et al. observed two major digestive *α-*amylases that were substantially (80 %) inhibited by the proteinaceous inhibitor α-AI1 from *Phaseolus vulgaris*, and could be a high-value target for coffee bean insect control using biotechnological strategies. The CBB’s *AmyHha* gene is primarily transcribed in the intestinal tract of *H. hampei* larvae (Bezerra et al. 2014). The very recent release of the CBB genome draft (Vega et al. 2015b) gives support to the role of the amylases in CBB digestion. The authors reported a wide variety of digestive proteinases of different classes likely to be capable of dealing with plant defensive proteins, which must probably turn challenging the control of CBB based on plant-produced proteinases. On the contrary, only one sequence matched to the *T. castaneum* α-amylase gene query. Besides, the CBB orthologous *α-*amylase gene presented high expression in females reared on a meridic diet in the laboratory, with an FPKM of 52997.

The use of plant-encoded genes functioning as bioinsecticides to produce insect-resistant transgenic crops has many potential benefits (Gatehouse and Gatehouse 1998; Silva et al. 2009; Lüthi et al. 2015). Endogenous amylase inhibition encoded by plant genes has been reported to cause nutrient deprivation in insect pests that attack economically important crops (Ishimoto et al. 1996; Mehrabadi et al. 2012).

Insect resistance via the adoption of GM crops has been highlighted as economically and agronomically advantageous versus conventional breeding approaches for farmers worldwide (Areal et al. 2013). Previously (Barbosa et al. 2010), we have demonstrated that crude seed extracts from genetically modified (GM) *C. arabica* plants expressing the α-amylase inhibitor-1 gene (*α-AI1*) under the control of the common bean *P. vulgaris* seed-specific promoter PHA-L inhibited 88 % of CBB α-amylases during *in vitro* assays, in which the *α-*AI1 protein constituted approximately 0.29 % of the crude seed extract. The presence of the *α-AI1* gene in the T1 generation plants was confirmed, and their germination rate was similar to that of the non-transformed plants, indicating that the transgene did not affect this phenotype.

The use of tissue-specific promoters is an important approach for increasing the yield of desired transgenic products by directly driving expression in the target tissue or organ. Seed-specific promoters can be used to target transgene expression specifically to grains, such as in rice, barley and wheat (Furtado et al. 2009). A recent review on the genetic transformation of coffee plants has reported that, currently, transgenic constructs for coffee plants almost exclusively use the constitutive CaMV35S viral promoter to introduce beneficial agronomic traits (Mishra and Slater 2012). After the sequencing of the complete coffee genome, a demand for promoters to drive tissue-specific gene expression in coffee plants has emerged (Denoeud et al. 2014).

To study the *in vivo* expression of the *α-AI1* gene driven by PHA-L in GM *C. arabica* plants, we characterized materials from six independent transformation events to evaluate regarding: i) the expression of *α-AI1* in different plant tissues, by RT-PCR of T1 lines representing three transformation events, ii) the localization of the α-AI1 protein in endosperm cells, by immunocytochemistry of mature fruits from T0 mother plants, iii) the segregation pattern of a single-copy event in the T2 progeny, by PCR analysis of 54 T2 individuals, and iv) CBB insect development in seeds from mature T2 fruits.

## Materials and Methods

### RNA Extraction

Total RNA was extracted from grain, leaf, stem and root tissues of GM *C. arabica* expressing α-AI1. Materials were collected from three PCR positive T1 lines derived from independent transformation events (T0 events 1, 2 and 3 and T1 analyzed by Barbosa et al. 2010). Materials from the T1 plants were pooled to form samples of each different tissue and samples were ground separately in liquid nitrogen. Approximately 30 mg of powder from each sample was processed using the RNAspin Mini RNA Isolation kit (GE Healthcare UK Limited, Buckinghamshire, UK) as follows: samples were transferred to a 1.5 mL sterile polypropylene tube to which 350 μL of buffer RA1 and 3.5 μL of β-mercaptoethanol were added. The sample was vigorously mixed, incubated for 10 min and centrifuged at 5,000 ×*g* for 1 min. The supernatant was transferred to an RNAspin Mini Filter and centrifuged at 11,000 x g for 1 min. Next, 350 μL of 70 % ethanol was added to the filtrate, and the mix was transferred to an RNAspin Mini Column for centrifugation at 8,000×*g*. Membrane desalting buffer (350 μL) was added to the column, followed by centrifugation at 11,000×*g* for 1 min. The filtrate was discarded, and 95 μL DNAse reaction mixture was added to the column. The column was washed once with Wash Buffer I and twice with Wash Buffer II. RNA was eluted with 100 μL of RNase-free H_2_O and centrifugation at 11,000×*g* for 1 min. RNA samples were stored at −80 °C*.*

### RT-PCR

cDNA was synthesized from 5 μg of total RNA from the pooled samples of each different tissue (grains, leaves, stems and roots) of T1 GM *C. arabica* plants expressing α-AI1 using the Superscript II First-Strand Synthesis System for RT-PCR kit (Invitrogen, California, USA). The presences of the α-AI1 and GAPDH genes were detected by RT-PCR using the following primers: α-AI1 forward (5’-GCCTTGGGATGTACACGA CT-3’), α-AI1 reverse (5’-CTCCATTGATAAGCCCCTGA-3’), GAPDH forward (5’-TTGAAGGGCGGTGCAAA-3’) and GAPDH reverse (5’-AACATGGGTGCAT CCTTGCT-3’). The GAPDH gene is a constitutive gene used as a positive control. The amplification reactions were performed under the following conditions: 5 min at 95 °C; 30 cycles of 45 s at 95 °C, 1 min at 60 °C and 30 s at 72 °C; and a final extension at 72 °C for 10 min. The resulting PCR product was separated on a 1 % agarose gel stained with ethidium bromide and visualized under a UV transilluminator.

### Segregation Analysis by PCR

Genomic DNA from the leaves of *C. arabica* non-transformed and T2 plants from event 2 (Barbosa et al. 2010) were purified via the CTAB method (adapted from Bernatzky and Tanksley 1986). Standard PCR experiments were performed (Bio-Rad T100 Thermal Cycler) to verify the presence of the *α-AI1* gene in the samples. The sequences of the primers used were 5'- GCCTTGGGATGTACACGACT-3' (forward) and 5'- CTCCATTGATAAGCCCCTGA-3' (reverse). The PCR reactions were performed in 20 μL containing approximately 100 ng of genomic DNA from the transformed plants (or non-transformed plants as a control), 1X buffer (CenBiot), 20 mM MgCl_2_ (Ludwig Biotec, Alvorada, Brazil), 4 mM DNTPs (Ludwig Biotec), 1 U of *Taq* DNA Polymerase (CenBiot), and 2.5 mM of each primer. PCR reactions were performed with an initial denaturation at 95 °C for 10 min; 36 cycles of 45 s of denaturation at 95 °C, 45 s of annealing at 60 °C and 1 min of extension at 72 °C; and a final 5 min extension step at 72 °C*.* The expected amplicons were 200 bp in length and were visualized on a 1 % agarose gel stained with ethidium bromide under UV light.

### Immunocytochemistry

Non-transgenic and transgenic mature coffee berries (cherry beans) of the T0 event 2 plant (Barbosa et al. 2010) were collected at the final maturation stage (approximately 180–210 days after flowering) (de Castro and Marraccini 2006). Fruits were sliced and fixed overnight in 0.5 % glutaraldehyde + 2 % paraformaldehyde in 0.2 M cacodylate sodium buffer at pH 7.0. Dehydration and embedding were performed as described by de Almeida et al. (2004). Material was dehydrated in a gradient ethanol series (15 %, 30 %, 50 %, 70 %, 85 % and 3 × 100 % for 2 h each except the 70 % step, which was incubated overnight and supplemented with 1 mM DTT). The samples were subsequently incubated with 50 % ethanol/50 % butyl-methyl methacrylate (BM- 4:1) overnight. The samples were then placed in 100 % BM supplemented with 1 mM DTT and 0.5 % BEE for 24 h under a UV lamp at −20 °C*.* Sections 3.5 to 5 μm thick were placed on poly-L-lysine-coated slides and allowed to dry on a hot plate at 60 °C*.* Slides were pre-incubated with a blocking solution of 1 % BSA in cacodylate buffer (centrifuged for 5 min). Next, the slides were incubated with a 1:300 solution of primary antibody rabbit anti-α-AI1 for at least 1 h at 37 °C in blocking solution (BSA 1 %) and then centrifuged for 5 min. The supernatant containing the primary antibody was then added to the slides and incubated overnight at 4 °C, followed by incubation at 37 °C for 1 h. The samples were rinsed twice with 50 mM piperazine-N,N'-bis (ethanesulfonic acid) (PIPES) buffer pH 6.9 for 15 min and incubated for 1 h with secondary antibody goat anti-rabbit Alexa 488 conjugate (Invitrogen) diluted 1:300 in blocking solution. Tissue sections were stained with DAPI (1 μg/mL) and mounted in 90 % glycerol. Images were recorded using a ZEISS Axiophot fluorescence microscope.

### Bioassay

A bioassay was performed with insects obtained directly from coffee fields. The cherry beans were collected from T2 lineage of the transformation event 2 (Barbosa et al. 2010), which showed the best expression of the inhibitor *α-AI1*. Coffee berries from the non-GM Catuaí Vermelho cultivar were used as a control. Each experimental unit consisted of one grain at 40 % humidity individualized in one vial and infested with one adult CBB female. The vials were incubated in a controlled growth chamber (27 °C ± 1 °C, HR at 75 % ± 5 %), and insect development was observed over time. The seeds were evaluated at 10, 14, 18, 22 and 26 days after infestation (DAI). The following developmental stages of the insects in the coffee beans were recorded: eggs, larvae of the first and second instar (L1 and L2), pre-pupae (PP), pupae and adults (Álvarez et al. 2001). For both treatments (GM and control), sixteen replicates per treatment during the evaluation time (32 seeds opened at each time point) were analyzed. Averages and confidence intervals for each stage and the total number of individuals for each experiment were estimated. The best function that explained the number of individuals per grain over time was recorded. Each date of assessment was compared to the control with a t-test (*p* = 0.05). SAS software was used for statistical analysis.

## Results

### *α-AI1* Gene Expression is not Detected Outside the Seed

**Fig. 1:**
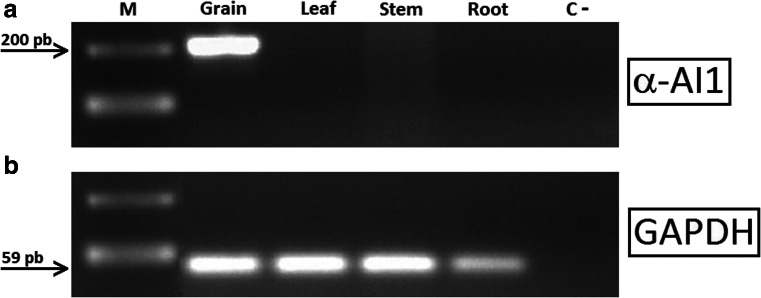
Organ-specific *α-AI1* gene expression in different tissues of GM *C. arabica.* RT-PCR profile visualized on 1 % agarose gels of grain, leaf, stem and root GM *C. arabica* cells using: **a** α-AI1- and **b** GAPDH-specific primers. Lane M: Marker – 100 bp ladder (Amersham Pharmacia Biotech Inc.); Lane C: negative control (no template); GAPDH: a constitutively expressed coffee gene serving as an endogenous positive control

To verify that the *α-AI1* gene is only expressed in seeds, we used RT-PCR to analyze *α-AI1* expression from cDNA samples synthesized from the mRNA present in the grains, leaves, stems and roots of GM *C. arabica* plants. The amplification results indicated the presence of the *α-AI1* transcript in the mRNAs extracted from the GM coffee seeds and the absence of this transcript in other organs, as shown by agarose gel electrophoresis (Fig. 1). The band corresponding to the *α-AI1* cDNA could only be detected in the grains, whereas the GAPDH constitutive gene (an endogenous control) was detected in all samples.

### The α-AI1 Protein is Present in the seed’s Endosperm

**Fig. 2:**
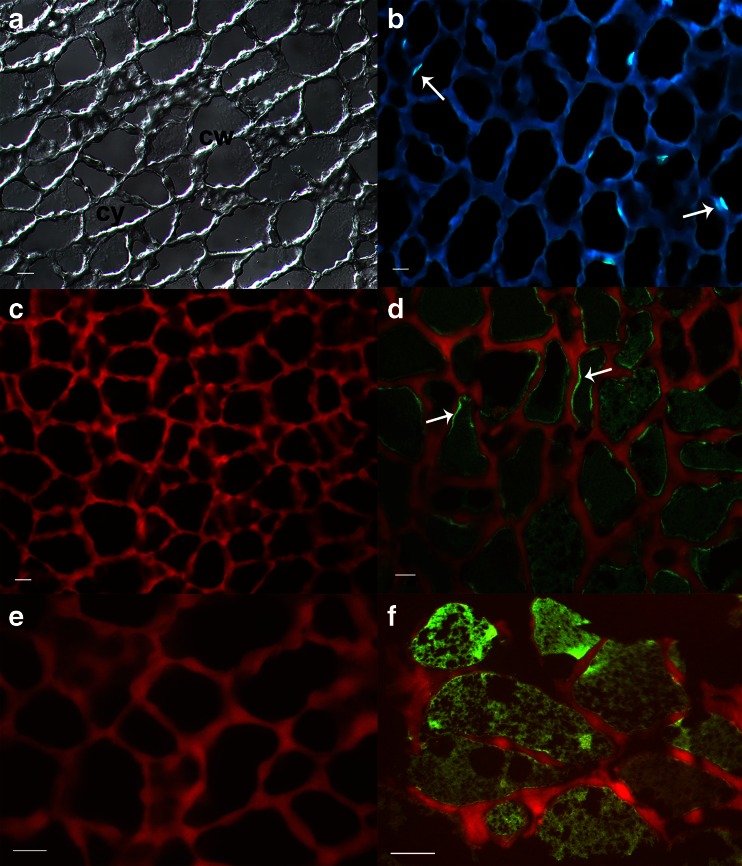
Micrographs of transverse sections of *Coffea arabica* endosperm. **a** DIC image visualizing the overall endosperm cell walls (cw) and cytoplasm (cy). **b** DAPI staining showing the bright signals that correspond to the nuclei close to cell walls (*arrows*). Fluorescence micrographs of the immunolocalization of the α-AI1 protein with anti-α-AI1 antibody in non-transformed (c and e) and transformed (d and f) endosperm, demonstrating the presence of the protein in the cytoplasm (green) and auto-fluorescence of the cell wall (red). The signal is often brighter along the cell wall (*arrows*) corresponding to the cytoplasm. The black inner cell regions are most likely oil droplets and are marked with asterisks (*). Bars indicate 20 μm

Because the insect feeds mainly on endosperm, it is important to verify that the α-AI1 protein is expressed in this particular seed tissue. Analysis of the GM fruit tissue sections illustrated the typical irregularly shaped endosperm cell walls as viewed by differential interference contrast microscopy (Fig. 2a). DAPI staining showed nuclei close to the cell walls, as indicated by arrows in Fig. 2b. The cell walls exhibited high auto-fluorescence when excited with UV light (red) using the Zeiss double bandpass filter 23 (Fig. 2c-f). α-AI-specific antibodies were visualized only in the presence of the α-AI1 protein in transgenic tissues (Fig. 2d and f), whereas non-transformed seeds were entirely devoid of a signal (Fig. 2c and e). Fluorescence, indicating the localization of the α-AI1 protein, was observed in endospermic cells of the transgenic plant. The intracellular signal was homogenously distributed in large central vacuoles comprising almost the entire cell and was brighter in the remaining exocentric cytoplasm at the periphery of the cell walls (indicated by arrows in Fig. 2d). Some spaces inside the vacuoles, indicated with asterisks in Fig. 2e, appear empty because they did not exhibit fluorescence. Similar spaces were identified as oil bodies (Acuña et al. 1999). The brighter signal appears to be distributed in the cytosolic part of the cytoplasm, but differently sized cytoplasmic spaces were not distinguishable, which suggests that the α-AI1 protein may be inside organelles.

### Amplification of the Transgene Shows Stable Expression of a Single Copy in T2 Plants

**Fig. 3:**
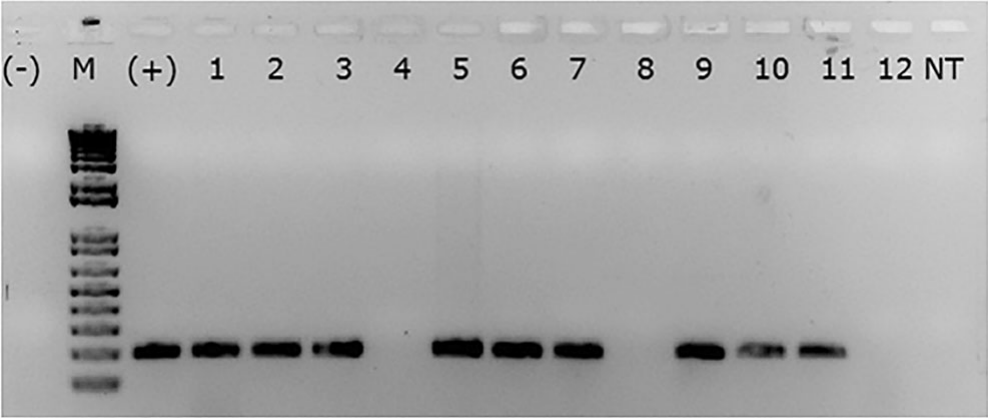
Segregation pattern of the *α-AI1* gene in T2 plants analyzed by standard PCR. PCR products from amplification of the transgene *α-AI1* in transformed and non-transformed plants of the T2 generation of *C. arabica.* **(−)**: reaction without template; **M**: 1 kbp Plus DNA Ladder (Invitrogen); lanes 1–12: transformed plants of *C. arabica*; **NT**: non-transformed plant

Approximately 54 plants from T2 progeny obtained from transformation event 2 (Barbosa et al. 2010) were tested by conventional PCR for the presence of the *α-AI1* gene. Samples were considered positive when the PCR produced an expected 200 bp-length amplicon, which corresponded to the expected size of the *α-AI1* gene (Fig. 3; sup Fig. 1). The results indicated an approximate segregation ratio in the T2 generation of 3:1 (3 possessing the transgene to 1 lacking the transgene), confirming that the transgene allele is dominant.

### The α-Amylase Inhibitor Affects CBB Development

**Fig. 4:**
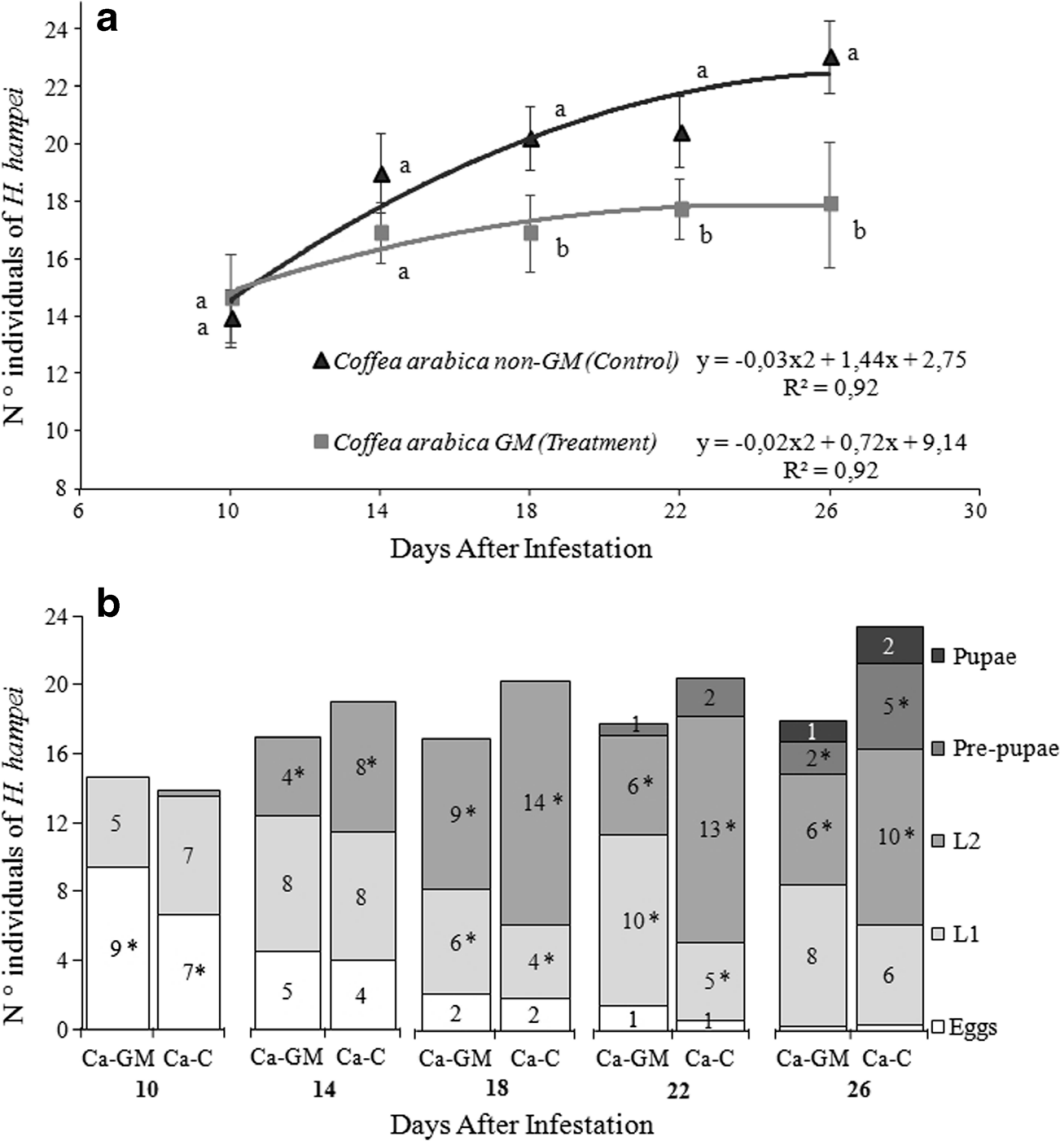
Bioassay of α-AI1-containing coffee fruits. Development of coffee berry borer insects (*H. hampei* (Ferrari)) reared on genetically modified (GM) *C. arabica* beans over time. **a** Total number of individuals after infestation. Different letters indicate statistically significant (*p* < 0.05) differences. Bars indicate 95 % confidence intervals. **b** Distribution graph of the immature stages of CBB. Ca-GM: genetically modified *C. arabica*. Ca-C: non-GM *C. arabica* (control). Asterisks (*) indicate significant differences between the number of individuals of the same stage for each date (*p* < 0.05)

Coffee berries expressing the α-AI1 protein were collected from the T1 transformed plants to test CBB development. The 26-day *in vivo* assay showed that fewer offspring developed when grown on GM berries than when grown on the non-transformed control grains (Fig. 4a). The consistently lower number of individuals emerging from the transformed grains, compared to the non-transformed control, suggested oviposition reduction effects on the adult females in the beginning of the infestation. Moreover, the statistical analyses for each stage showed significant differences in the number of eggs at 10 DAI, the number of L2 at 14 DAI, the number of L1 and L2 at 18 and 22 DAI, and the number of L2 and PP at 26 DAI (Fig. 4b). As the number of L1 increased over time in the transformed grains, there was a corresponding decrease in the number of L2 and PP, which suggests a delay in the CBB life cycle, likely due to the adverse nutritional effects of α-amylase inhibition on the insect life cycle.

## Discussion

A recent review Sharma (2012) highlighted the need to exploit modern biotechnology tools, such as genetic engineering and gene pyramiding, to increase host plant resistance levels to insect pests for sustainable pest management, crop protection and environmental conservation. These strategies for biotechnology-based pest management represent an attractive approach to obtaining a more sustainable agriculture based on transgenic crops (Duke 2011; Kamle and Ali 2013).

Transgene expression in *C. arabica* fruits was first reported using the GUS protein driven by the constitutive CaMV35S promoter (Albuquerque et al. 2009). Although constitutive promoters are now widely used, they are not suitable for all transgenes, especially for stress-responsive genes, where they can have serious deleterious effects. A recent study (Perthuis et al. 2015) demonstrated that the constitutive promoter *EF1α-A1* was negatively correlated with the nutritional status of the coffee plants and that the Cry1Ac protein levels in the transgenic leaves were too low to provide efficient and sustainable protection against *Leucoptera coffeella* in the field.

New frontiers for second- and third-generation transgenic plants involve tissue-specific expression driven by specific promoters (Christou et al. 2006). In a transgenic tobacco root assay, the promoter of a putative peroxidase-encoding gene from *C. arabica* (CaPrx) driving β-glucuronidase (GUS) expression was active in galls and was induced by root-knot nematode infection after 16 h (Severino et al. 2012). Recently, promoter regions from an *nsLTP* (non-specific lipid-transfer protein) type II gene that is specifically expressed in coffee fruits were reported to promote grain-specific expression in transgenic tobacco plants when driving GUS expression, as observed by histochemical and fluorometric GUS assays (Cotta et al. 2014).

In the present study, important experimental data were generated to characterize the expression pattern of the seed-specific promoter PHA-L in coffee grains. Offspring from the GM coffee plants reported by Barbosa et al. (2010) were cultivated for several years under greenhouse conditions to enable *in vivo* studies on the heredity, stability and expression of the *α-AI1* gene controlled by the PHA-L promoter. The materials used derived from six T0 independent events, T1 lines from events 1, 2 and 3, and T2 generation of the transformation event 2 (showing the best expression level and *in vitro* inhibition activity).

Our data strongly suggest that the transcription of the *α-AI1* transgene in coffee seeds is tissue-specific. The *α-AI1* mRNA was detected only in grains, as expected from the control of the seed-specific promoter PHA-L (Altabella and Chrispeels 1990). PCR detection of the transgene in T2 plants revealed segregation patterns that confirm the single-copy event observed previously (Barbosa et al. 2010) by Southern Blot analysis, in which only one band was hybridized with an entire [α-^32^P] dCTP probe. The zygosity estimation derived from PCR analysis on DNA extracted from leaves of the T2 plants confirmed the Mendelian inheritance pattern of a single-copy insertion, in which the transgene was present in three-quarters of these plants. Based on these results, we can infer that the PCR-positive individuals contain one or two copies of the α-AI1 gene. The endosperm of *C. arabica* plants with 2*n* = 44 is initially a triploid tissue, presenting groups of cells of different ploidy (Medina 1965), as in other plants (Vijayaraghavan and Prabhakar 1984) with a non-sporophytic origin (de Castro et al. 2001). The presence of the transgene was detected by PCR in the T1 progeny (Barbosa et al. 2010). Considering the complete self-pollination of a single-copy GM *C. arabica*, we may infer that at least one copy of the transgene is expected to be present in the triploid endosperm of the T2 beans used in the bioassay. The bioassay results show a clear tendency of the presence of the α-AI1 protein to influence the life cycle of the CBB by decreasing the oviposition rate and compromising the molting stages. The number of larvae in the L1 stage significantly outnumbered the number in the L2 stage at 18 and 22 DAI when comparing GM grains to control grains. Inversely, the number of L2 developing in GM grains was consistently higher than in the control from 14 DAI to 26 DAI, and the number of PP was significantly lower in GM grains at 26 DAI. Additionally, the total number of individuals in all collected points after 14 DAI was significantly lower in the GM treatments compared to the control treatments.

The α-AI1 expression level observed in transgenic coffee was slightly lower compared to levels observed in other transgenic plants containing this α-amylase inhibitor. The α-AI1 expression level in coffee reached a maximum of 0.29 % in fruits from T0 plants (Barbosa et al. 2010) and a mean of 0.14-0.16 % (sup table 1) in fruits from 4 lines of T1 plants (varying from 0.02-0.29 %). A low level of inhibitor expression (0.2 %) conferred protection against the pea weevil in field trials in transgenic pea (Morton et al. 2000). Higher α-AI1 expression levels were observed in transgenic chickpeas and pea seeds: 1.0-3.5 % in peas (Schroeder et al. 1995) and 4.2 % in chickpeas (Sarmah et al. 2004). In transgenic chickpeas, partial resistance to *Callosobruchus chinensis* was associated with a lower level of expression of α-AI1 (0.63-0.72 %) in some transgenic lines (Lüthi et al. 2013). Recombinant purified proteins were assayed in meridic coffee-based diet supplemented with chitinases (Martínez et al. 2012). Although there are several studies on CBB biology, no controlled artificial diet with determined contents of reagents for the CBB has been developed, which constitutes a major constraint for performing nutritional deprivation research on this insect (Brun et al. 1993). The strategy of developing resistant plants that inhibit the CBB’s digestive enzymes assumes that the CBB depends on the starch present in coffee grain polysaccharides. As CBB *a*-amylase activity is substantially inhibited (80 %) by relatively low levels of α-AI1, it was assumed that incorporation of the *a-AI1* gene into the coffee genome would confer substantial protection against CBB attack (Valencia et al. 2000). However, our bioassay may indicate that the starch is not vital to CBB development, as we verified no mortality effect *in vivo* by feeding insects on α-AI1-expressing seeds. The nutritional requirements of the CBB are barely known. The starch levels in coffee are considerably lower than in common beans. Variations in the starch content to evaluate to germination and plant conversion in different *C. arabica* cultivars show starch quantification of 20 mg/g fresh matter (Giorgini et al. 1992) or 30 mg/g dry weight (Etienne et al. 2013).

Starch in coffee seeds is present mainly in the embryo and cotyledons (Etienne et al. 2013), but coffee seeds contain other sugars that can be used as a carbon source, such as sucrose, glucose, mannose, fructose and many others (Murkovic and Derler 2006). During the inhibition of CBB amylase activity, these alternative sugars may be used as a carbon source. Galactomannan is another abundant polysaccharide encountered in the cell walls of *C. arabica*’s endosperm (Sutherland et al. 2004), and mannase hydrolysis was recently reported as a probable source for CBB nutrition (Acuña et al. 2012). Accordingly, the simultaneous inhibition of mannose and amylase activities through transgene pyramiding might constitute an even better strategy to control CBB attack. Another promising tool that could be used to confer CBB resistance is a proteinaceous inhibitor that was isolated from *Lupinus bogotensis* seeds, which showed effective biological activity against aspartic proteases (Molina et al. 2010), digestive proteases that are also present in the CBB intestinal tract. Transgene pyramiding has been reported to positively affect insect control in cotton, rice, cabbage and other crops (Patel et al. 2013; Yi et al. 2013; Xu 2013).

Immunolocalization successfully revealed the presence of the α-AI1 protein in the endosperm of transformed *C. arabica* plants as well as its absence in the same tissue of non-transformed plants. In this case, the *α-AI1* gene driven by the common bean PHA-L promoter was used to genetically transform coffee. The post-transcriptional processing of the α-AI1 protein in *P. vulgaris* tissue includes the removal of a signal peptide, passage through the endoplasmic reticulum and Golgi apparatus, and subsequent transport of the protein into storage vacuoles (Campbell et al. 2011). The same post-transcriptional processing likely occurs with the α-AI1 protein in the GM *C. arabica* fruits. We observed that the α-AI1 protein was present in the cytoplasm and central vacuole of the GM coffee endosperm cells; similarly, the storage proteins glycinin and legumin were detected by immunogold-labeling in *P. vulgaris* cotyledons and in coffee endosperm (Acuña et al. 1999), respectively, in storage vacuoles and in the cytoplasm. We observed a broad signal in the central part of the GM coffee endosperm cell and a brighter fluorescence close to the cell wall. These findings indicate that α-AI1 is mainly confined to vacuoles but also accumulates in the cytoplasm, as was observed for other storage proteins. Although the organelles are not clearly distinguished in the cytoplasm surrounding the central vacuole at the developmental stage observed in the coffee GM fruits, the brighter signal close to the cell wall may indicate that α-AI1 proteins in coffee seeds are subject to the secretory pathway.

The CBB is an important coffee crop pest due to its worldwide distribution and its restricted development within the coffee berry (Vega et al. 2009). The results presented here may provide tools to better control this insect pest, as the application of biotechnology could greatly reduce costs and the use of agrochemicals to increase the yield of coffee. The containment of transgene expression to the fruit with a seed-specific promoter is also beneficial for biosafety; because the α-AI1 protein is denatured at high temperatures (Bezerra 2013), we assume that the inhibitor will be safe for human consumption if the expression is directed toward the grain, which is roasted before beverage preparation. Additionally, contact between transgene products expressed in the grain and non-target organisms present on roots and leaves is highly minimized.

### Final Considerations

The present study demonstrates that the PHA-L promoter can be used to drive seed-specific transgene expression in coffee grains. This specificity should be a valuable resource for transgene containment in biotechnological approaches to coffee plant improvement. It is interesting to note that the by PHA-L conferred in coffee similar ectopic localization of the protein observed in legumes, despite the fact that *C. arabica* is a woody shrub and most of its grain is constituted of endosperm, whereas the grains of the common bean, chickpea and pea are mostly composed of cotyledon.

The *α-AI1* transgene under the control of the PHA-L promoter was stably passed to the T2 progeny. Moreover, no PHA-L was detected in other parts of the coffee plants. The homogeneous expression pattern of the α-AI1 protein in the endospermic cells indicates that the insect is likely to ingest the inhibitor when feeding on the transgenic seed.

The *in vivo* effect of *α-AI1* expression on CBB development was less than expected, given the inhibition previously observed *in vitro*. Further experiments should be conducted with homozygous plants in the field to assess potential deleterious effects on insect development and reduction in insect progenies.

## Electronic supplementary material

ESM 1 (DOCX 145 kb)

ESM 2 (DOCX 13 kb)
