# Supplementary material for: Seed-Specific Stable Expression of the α-AI1 Inhibitor in Coffee Grains and the In Vivo Implications for the Development of the Coffee Berry Borer
Source: Trop Plant Biol. 2015 Oct 8;8:98–107. doi: 10.1007/s12042-015-9153-0 (PMC4676793; doi:10.1007/s12042-015-9153-0)
Supplement: Supplementary file 1 — (DOCX 145 kb) [file 12042_2015_9153_MOESM1_ESM.docx]

a


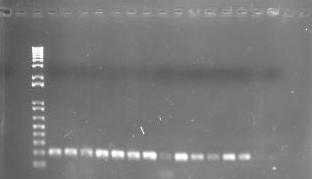


(-) M (+) 1 2 3 4 5 6 7 8 9 10 11 12 - NT NT’

b


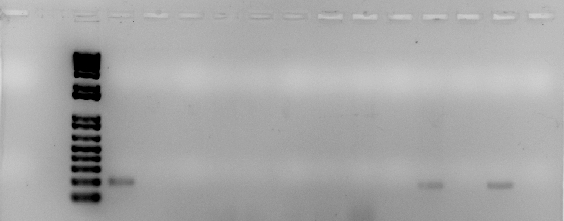


NT (-) M (+) 1 2 3 4 5 6 7 8 9 10 11 12

c


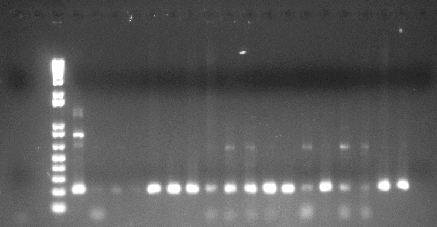


(-) M (+) 1 2 3 4 5 6 7 8 9 10 11 12 13 14 15 16 17 NT

**Fig 1S**: Segregation pattern of the *α-AI1* gene in T2 plants analyzed by standard PCR. PCR products from amplification of the transgene *α-AI1* in transformed and non-transformed plants of the T2 generation of *C. arabica.* DNA samples from the progeny in (a) **lanes 1-12**, (b) **lanes 1-12**, and (c) **lanes 1-17**; **(-)**: reaction without template; **M**: 1 kbp Plus DNA Ladder (Invitrogen); **NT**: non-transformed plant.
