# Supplementary material for: Seed-Specific Stable Expression of the α-AI1 Inhibitor in Coffee Grains and the In Vivo Implications for the Development of the Coffee Berry Borer
Source: Trop Plant Biol. 2015 Oct 8;8:98–107. doi: 10.1007/s12042-015-9153-0 (PMC4676793; doi:10.1007/s12042-015-9153-0)
Supplement: Supplementary file 2 — (DOCX 13 kb) [file 12042_2015_9153_MOESM2_ESM.docx]

Sup Table 1: α-AI1 expression analysis by Elisa of T2 fruits from GM coffee beans used in this work to the bioassays with the coffee berry borer (Hypothenemus grandis). The table shows in (d) the average of the grain protein expression of each T1 plant (a) from seeds collected (one a day) after the bioassays on different days (10, 14, 18, 22 and 26) of incubation. Results of the detection of the α-AI1 by Elisa in (b) were correlated to the total protein (TP) measured by Bradford method in (c), and the Standard Deviation (SD) calculated in (e).

| **^a^ Plant (T1)** | **^b^ mean % α-AI1** | **^c^ mean** αAI-1 (µg/µL) | **^d^ mean TP (**µg/µL) | **^e^ SD % αAI-1** |
| --- | --- | --- | --- | --- |
| 1 | 0,138 | 0,001 | 0,773 | 0,038 |
| 2 | 0,157 | 0,001 | 0,698 | 0,079 |
| 3 | 0,146 | 0,001 | 0,680 | 0,059 |
| 4 | 0,149 | 0,001 | 0,650 | 0,094 |
